# Serum lipid concentrations in patients with cholesterol and pigment gallstones

**DOI:** 10.1186/1756-0500-7-548

**Published:** 2014-08-19

**Authors:** Harshi Thilanka Welegedara Weerakoon, Shirani Ranasinghe, Ayanthi Navaratne, Ramaiah Sivakanesan, Kuda Banda Galketiya, Shanthini Rosairo

**Affiliations:** Department of Biochemistry, Faculty of Medicine and Allied Sciences, Rajarata University of Sri Lanka, Saliyapura, Sri Lanka; Department of Biochemistry, Faculty of Medicine, University of Peradeniya, Peradeniya, Sri Lanka; Department of Chemistry, Faculty of Science, University of Peradeniya, Peradeniya, Sri Lanka; Department of Surgery, Faculty of Medicine, University of Peradeniya, Peradeniya, Sri Lanka; Department of Radiology, Faculty of Medicine, University of Peradeniya, Peradeniya, Sri Lanka; Postgraduate Institute of Science, University of Peradeniya, Peradeniya, Sri Lanka

**Keywords:** Cholesterol gallstones, Pigment gallstones, Lipid profile

## Abstract

**Background:**

Gallstones (GS) are formed as a result of impaired metabolic regulation of the human body. Abnormal lipid metabolism is partly responsible for the pathogenesis of GS mainly rich in cholesterol. Thus abnormalities of serum lipids would reflect the possibilities of the formation of cholesterol GS. This study aims to identify the significance of serum lipids on the development of GS disease.

**Methods:**

Serum lipid profiles were estimated in 73 patients with symptomatic GS, admitted to the Teaching Hospital, Peradeniya, Sri Lanka for GS removal surgeries from May 2011 to December 2012. Patients with normal serum bilirubin level and not being on lipid lowering drugs were recruited for the study. Serum lipid profile of each patient was analyzed by enzymatic kit assays and the chemical composition of GS was analyzed by Fourier Transform Infrared spectroscopy.

**Results:**

Of the 73 patients, 37 (51%) had cholesterol GS while 36 (49%) had pigment GS. Serum lipid parameters of a majority of patients were within the normal range. Body mass index values of the patients with two types of GS were not significantly different (Two sample *t* test, p = 0.335). Out of the lipid parameters tested, only serum triglyceride concentration was significantly high in patients with cholesterol GS than that of pigment GS (Two sample *t* test, p = 0.038). None of the lipid parameters were significantly different between males and females (Two sample *t* test, p > 0.05). Compared to the patients with pigment GS who were aged below 50 years, mean total cholesterol and triglyceride concentrations were higher in the same age category patients with cholesterol GS.

**Conclusion:**

Abnormal serum lipid profiles doesn’t seem to be an essential feature in patients with cholesterol GS. However when the two groups of patients with cholesterol and pigment GS with no significant difference of body mass indexes were compared, patients with cholesterol GS are more likely to have serum lipid parameters towards the undesirable cutoff levels of their respective normal ranges. However an effect of serum lipid concentrations on high incidence of GS among females has not been identified.

## Background

Gallstones (GS) are formed in the gall bladder and biliary tract and are of two types: namely cholesterol and pigment stones [1]. GS is one of the main causes for number of upper gastrointestinal surgical casualties [2]. Despite the wider exploration into the aetiopathogenesis of GS, knowledge on exact pathogenesis of GS is yet incomplete. Complex interaction of multiple environmental risk factors with the genetic risk factor is the most possible explanation given for this incomplete understanding. Biliary cholesterol supersaturation is identified as a main prerequisite for the formation of cholesterol GS [3] and elevated unconjugated bilirubin in bile is considered as the primary cause for the pigment GS [4]. Thus GS are formed as a result of impaired metabolic regulation of human body. Biliary cholesterol hypersecretion is the main cause for biliary cholesterol supersaturation and bile stasis also plays an additional role [5]. Impaired lipid homeostasis can give rise to cholesterol hypersecretion from biliary canaliculi. Therefore high incidence of cholesterol GS compared to pigment GS can be expected in patients with impaired lipid homeostasis.

Even though a positive correlation between serum triglycerides (TG) and nucleation time of cholesterol in bile is identified, a relationship between serum lipids and biliary cholesterol saturation index has not been identified [6]. Moreover, controversial results have been obtained for the serum lipid profiles of patients with GS. In some case control studies, serum hypertriglyceridemia and low HDL-cholesterol (HDL-C) have shown a significant association with GS disease [7–9]. However it has not been a common observation [10].

If impaired lipid homeostasis is the main cause for cholesterol supersaturation and crystallization, a significant difference between serum lipid profiles of patients with cholesterol and pigment GS should be observed, as cholesterol hypersecretion is not a recognized cause for the pathogenesis of pigment stones. Therefore comparison of lipid profiles in patients with two types of GS would be important in detecting the significance of different lipid parameters on the development of compositionally different GS. Moreover serum lipids might have an effect on the high incidence of GS disease among females [11] and on the age related changes in the chemical composition of GS [12]. The objective of this study was to identify the relationship between serum lipids and the pathogenesis of different types of GS.

## Methods

The study was carried out during May 2011 to December 2012 in a cohort of patients with symptomatic GS, admitted to Teaching hospital, Peradeniya, Sri Lanka for surgical removal of GS. Inclusion criteria for the study were, age more than 18 years, residing in Kandy district, Sri Lanka for more than five years, normal total bilirubin concentration [13] and not being on lipid lowering drugs. Informed written consent was taken from the patients at the time of recruitment. An interviewer administered formatted data sheet which included age, gender, comorbidities and drug history of the patient was used in data collection. Weight and height of each patient was measured using standard techniques [14] to calculate the body mass index (BMI). According to BMI values, patients were classified into two groups as normal and overweight or obese based on the WHO BMI cutoff values [15]. A sample of 2 ml venous blood was taken from each patient after a fasting period of 12 hours [16]. Serum samples were stored at -20°C and the lipid profiles were analyzed within 4 weeks of sample collection. Total Cholesterol (TC), TG and HDL- C in serum were analyzed by enzymatic methods using enzyme assay kits purchased from Aggape Diagnostics, Germany. All the tests were done in duplicates and mean value was taken as the end result. Measurements of the standard samples were taken at the beginning, middle and end of the test samples to improve the accuracy of the readings. LDL- cholesterol (LDL-C) was calculated using Friedewald equation [17].

Gallstones removed at the time of surgery were preserved appropriately and the chemical composition was identified by the physical characteristics and Fourier Transform Infrared spectroscopy (FTIR, Shimadzu IR prestige 21). Previously described methods were used in the analysis of chemical composition of the GS by FTIR [18, 19]. GS were divided in to two groups as, cholesterol and pigment GS, based on the chemical composition [1] and the lipid profile results of the two groups were compared in the final analysis. In addition, serum lipid profiles of patients with the two types of GS were compared after categorizing them in to two age groups each as 50 years or less and above 50 years. This was done based on the median age of menopause of Sinhalese females [20].

Data obtained were analyzed by Minitab 14 software in which two sample *t* test was used to analyze the continuous data, while chi square and Fisher’s exact tests were used in the analysis of categorical data.

The cutoff values used to determine the desirable levels of serum lipids were; serum TC below 200 mg/dl, TG below 150 mg/dl, HDL-C above 40 mg/dl and LDL-C below 100 mg/dl [21]. Ethical clearance for the study was obtained from the Ethical Review Committee, Faculty of Medicine, University of Peradeniya, Sri Lanka.

## Results

A total of 73 patients were recruited to the study, based on the inclusion criteria. Mean age of the total study group was 44.6 ± 10.4 years and majority was females. (*n* = 60, 82%). Mean ages of females and males were 44.0 ± 10.4 and 47.1 ± 10.0 years respectively. A greater proportion (*n* = 52, 71%) of patients in the study group were aged 50 years or less.

Of the total, 37 (51%) had cholesterol GS while 36 (49%) had pigment GS. Age and gender distribution of patients with two types of GS are shown in Table 1. Mean age of the patients with cholesterol GS was 41.1 ± 9.2 years and those with pigment GS was 48.5 ± 10.3 years, denoting a significant difference (Two sample *t* test, p = 0.002).

**Table 1:** **Age and gender distribution of patients with two types of GS**

Physical parameter	Cholesterol GS	Pigment GS	P value
Age (mean ± SD) years	41.1 ± 9.2	48.5 ± 10.3	0.002*
Gender (*n*,%)			
Females	34 (57%)	26 (43%)	0.035^$^
Males	03 (23%)	10 (77%)	

*-Two sample *t* test.

^$^-Fisher’s exact test.

Among the 60 females, majority (*n* = 34, 57%) had cholesterol GS, while majority of males (*n* = 10, 77%) had pigment GS. Compared to males the cholesterol GS were common among females (Fisher’s exact test, p = 0.035).

In the group of patients aged ≤ 50 years, majority (*n* = 31, 60%) had cholesterol stones whereas majority (*n* = 15, 71%) of patients aged > 50 years had pigment stones. Frequency of detecting cholesterol GS among the patients aged ≤50 years was significantly common compared to that of patients aged > 50 years (Chi square test, p = 0.010). Mean BMI of the patients with cholesterol GS (26.06 ± 3.15 kg/m^2^) was not statistically significant (Two sample *t* test, p = 0.335) from that of the patients with pigment GS (25.28 ± 3.28 kg/m^2^). Mean concentrations of serum total cholesterol, TG, HDL-C and LDL-C in the total study group were 169.9 ± 27.6, 149.1 ± 24.7, 52.9 ± 9.6 and 87.2 ± 30.7 mg/dl respectively.

Even though none of the patients in this study cohort had a history of dyslipidemia few had serum lipid parameters in undesirable range (Table 2). Serum lipid concentrations in patients with cholesterol and pigment GS are shown in Table 3. In the comparison of serum lipids among patients with two types of GS, serum TG, TC and HDL-C were higher in patients with cholesterol GS than pigment GS. However a statistical significance was seen only for serum TG (Two sample *t* test, p = 0.038).

**Table 2:** **Undesirable lipid parameters in patients with cholesterol and pigment GS**

Serum lipid parameter	No of patients in undesirable level	
	Cholesterol GS *n*(%)	Pigment GS *n*(%)
TC	07 (19)	03 (08)
TG	05 (14)	03 (08)
HDL-C	00 (00)	03 (08)
LDL-C	07 (19)	12 (33)

**Table 3:** **Comparison of serum lipids in patients with cholesterol and pigment GS**

Serum lipid parameter (mg/dl)	Cholesterol GS (*n* = 37) Mean ± SD	Pigment GS (*n* = 36) Mean ± SD	P value*
TC	172.0 ± 30.1	167.8 ± 25.1	0.515
TG	155. 0 ± 20.9	143.00 ± 27.1	0.038
HDL-C	54.05 ± 9.19	51.5 ± 10.0	0.261
LDL-C	86.9 ± 33.0	88.5 ± 29.0	0.826

*Two sample *t* test.

Serum lipids among male and female patients are shown in Table 4. None of the serum lipid parameter was significantly different between females and males (Two sample *t* test, P > 0.05).

**Table 4:** **Comparison of serum lipids in females and males in the total study group**

Serum lipid parameter (mg/dl)	Females (*n*=60) Mean ± SD	Males (*n*=13) Mean ± SD	P value*
TC	171.1 ± 28.8	164.7 ± 21.6	0.374
TG	149.4 ± 23.8	147.9 ± 29.6	0.866
HDL-C	53.82 ± 8.39	48.11 ± 9.64	0.065
LDL-C	87.8 ± 32.0	87.0 ± 26.0	0.924

*Two sample *t* test.

Serum lipid profiles in patients with two types of gallstones were further analyzed after categorizing them into two age groups as age ≤ 50 years and > 50 years and are summarized in Tables 5 and 6. Irrespective of the age category high serum TC and TG were seen among the patients with cholesterol GS. However none of these parameters were significantly different (Two sample *t* test, p > 0.05) in both age categories.

**Table 5:** **Comparison of serum lipids in patients aged ≤ 50 years with two types of GS**

Serum lipid parameter (mg/dl)	Cholesterol GS (*n* = 31) Mean ± SD	Pigment GS (*n* = 21) Mean ± SD	P value*
TC	171.10 ± 32.0	165.1 ± 22.4	0.431
TG	153.50 ± 20.2	141.00 ± 30.7	0.108
HDL-C	54.86 ± 9.50	56.08 ± 9.34	0.650
LDL-C	85.50 ± 34.5	80.80 ± 26.2	0.581

*Two sample *t* test.

**Table 6:** **Comparison of serum lipids in patients aged > 50 years with two types of GS**

Serum lipid parameter (mg/dl)	Cholesterol GS (*n* = 6) Mean ± SD	Pigment GS (*n* = 15) Mean ± SD	P value*
TC	176.94 ± 17.94	171.57 ± 28.80	0.615
TG	162.70 ± 24.9	145.97 ± 21.76	0.188
HDL-C	51.29 ± 7.16	45.13 ± 6.65	0.107
LDL-C	93.12 ± 21.12	97.25 ± 30.98	0.731

*Two sample *t* test.

## Discussion

This study describes the pattern of serum lipid concentrations in a cohort of patients with cholesterol and pigment GS. Further in this study cohort, BMI values of the patients with two types of GS were not significantly different. Despite that serum TG and TC were high among the patients with cholesterol GS compared to that of pigment GS. Of these two parameters, serum TG showed a significant difference as well. Further serum TG and TC concentrations were high even in young patients with cholesterol GS compared to that of pigment GS. This signifies the possible involvement of serum TG and TC on the development of cholesterol GS even in young patients. Moreover, none of the lipid parameters were significantly different between females and males. As the study was carried out among the patients with GS disease with no prior diagnosis of dyslipidaemia, the lipid parameters were normal in majority of patients. Findings similar to the current study have been observed among Indian patients with GS disease [22] and this signifies the possibility of developing GS even in people with normal lipid parameters. Detection of significantly high serum TG in patients with cholesterol GS is a supportive evidence to indicate hypertriglyceridemia as a risk factor of cholesterol GS [23]. High serum TG in patients with GS is revealed in case control studies carried out in countries with high prevalence of GS disease as well [7–9]. The current finding further illustrates the significant role of serum TG in the determination of chemical composition of GS among the stone formers. Hypertriglyceridemia is identified as a cause for gall bladder (GB) hypomotility as it reduces the GB sensitivity to cholecystokinin [24], a paracrine hormone that regulates the GB contraction. GB hypomotility is one of the main causes for cholesterol crystallization [25]. Therefore the negative effect of serum TG on GB motility can be considered as a cause to develop cholesterol GS than pigment GS in people with high risk of GS disease.

The prevalence of GS depends on the gender where females have a higher incidence [11]. Multiple reasons have been evaluated in the identification of the cause for this gender difference. However a possible effect of serum lipids causing high incidence of GS among females was not identified in this study. Interestingly female patients in the study group had high serum HDL-C than that of male patients. The favorable effects of female reproductive hormones on serum lipids can be considered as a possible reason for such observation, as mean age of females in the study group was below the menopausal age which is considered as 51 years for Sinhalese [20]. However, a high risk of GS among patients with low HDL-C has been observed [8].

In this study cohort, patients with pigment GS were significantly older than those with cholesterol GS. In spite of this, the serum lipid abnormalities were more common among patients with cholesterol GS showing the possibilities of developing cholesterol GS even in young patients with impaired serum lipids. Importantly high serum TG concentrations even at younger ages can be considered as a risk factor to have cholesterol GS. Moreover, aging is associated with significant changes in lipid profile and as well as in the chemical composition of GS. High incidence of pigment GS is observed in elderly population as cholesterol content in GS gradually reduces with aging [12]. Similar changes in the GS composition were seen in patients in this study cohort as well. Though cholesterol GS was significantly high among patients aged ≤ 50 years, none of the lipid parameters were significantly different between young (age ≤ 50 years) and elderly patients (age > 50 years) in the two types of GS. Though it was not statistically significant, the results obtained for serum lipids for cholesterol GS patients aged ≤ 50 years indicate a higher tendency to have abnormal lipid profiles in their future lives than that of pigment GS patients. Importantly the lipid profile pattern seen among these patients should be evaluated with caution as impaired lipid homeostasis is a main risk factor for the cardiovascular disease as well [21]. Thus the development of GS disease can be considered as a warning sign to have high risk of cardiovascular disease for the patients with cholesterol GS. Increased mortality due to cardiovascular disease among patients with GS in USA has been observed [26].

In the current study patients with cholesterol GS aged > 50 years had high serum HDL-C compared to that of patients pigment GS and such finding was not observed among the patients aged ≤ 50 years. However, the presence of high serum TC and TG among cholesterol GS patients in both age categories signifies the effect of serum TC and TG on the formation of cholesterol GS over pigment GS irrespective of the age.

## Conclusion

Abnormal serum lipid profiles doesn’t seem to be an essential feature in patients with cholesterol GS. However when the two groups of patients with cholesterol and pigment GS with no significant difference in body mass indexes were compared, patients with cholesterol GS are more likely to have serum lipid parameters towards the undesirable cutoff levels of their respective normal ranges. However an effect of serum lipid concentrations on high incidence of GS among females has not been identified.

## Abbreviations

BMI: Body mass index
GB: Gallbladder
GS: Gallstones
HDL-C: HDL cholesterol
LDL-C: LDL cholesterol
TC: Total cholesterol
TG: Triglycerides.
